# Complete Genome Sequence of Lactobacillus paracasei EG9, a Strain Accelerating Free Amino Acid Production during Cheese Ripening

**DOI:** 10.1128/genomeA.00627-18

**Published:** 2018-07-05

**Authors:** Yui Asahina, Akino Shiroma, Kazuma Nakano, Hinako Tamotsu, Noriko Ashimine, Misuzu Shinzato, Maiko Minami, Makiko Shimoji, Tetsuhiro Nakanishi, Shun Ohki, Kuniko Teruya, Kazuhito Satou, Miho Kobayashi, Tatsuro Hagi, Naoko Moriya, Chise Suzuki, Atsushi Tajima, Masaru Nomura, Takashi Hirano

**Affiliations:** a Graduate School of Life and Environmental Sciences, University of Tsukuba, Tsukuba, Ibaraki, Japan; b Institute of Livestock and Grassland Science, NARO, Tsukuba, Ibaraki, Japan; c Okinawa Institute of Advanced Sciences, Uruma, Okinawa, Japan

## Abstract

Lactobacillus paracasei EG9 is a strain isolated from well-ripened cheese and accelerates free amino acid production during cheese ripening. Its complete genome sequence was determined using the PacBio RS II platform, revealing a single circular chromosome of 2,927,257 bp, a G+C content of 46.59%, and three plasmids.

## GENOME ANNOUNCEMENT

Lactobacillus paracasei belongs to the Lactobacillus casei group, along with L. casei and L. rhamnosus, and it inhabits various environments, such as plants, fermented foods, and the intestinal tract of humans and animals (1). L. paracasei is widely accepted as one of the dominant species of nonstarter lactic acid bacteria involved in cheese ripening (2, 3). As it influences ripening and flavor development (2–5), attempts have been made to utilize L. paracasei as an adjunct starter in cheese ripening (6–8).

L. paracasei EG9 was isolated from well-ripened cheese (9). In a cheese ripening environment at 10°C in the presence of 1.7% NaCl, the growth of EG9 is not suppressed, in contrast to that of the type strain L. paracasei subsp. paracasei JCM 8130. The addition of EG9 to lactic fermentation starter increased the total free amino acid content of the cheese. Based on these results, EG9 was considered a potential adjunct starter in accelerating cheese ripening. To identify potential genetic determinants that specify the properties of strain EG9, we sequenced the whole genome using single-molecule real-time (SMRT) technology (10). SMRT technology offers advantages, such as long read lengths, high consensus accuracy, and a low degree of bias, and is a powerful tool for sequencing and assembling complete bacterial genomes containing highly repetitive sequences (11, 12).

The genomic DNA was purified from cells in the early log phase using a PowerClean DNA cleanup kit (Mo Bio Laboratories, Carlsbad, CA). This was followed by the construction of a 20-kb library for P6-C4 chemistry with shearing (12). Size selection was not performed. Seven SMRT cells (a 240-min movie per cell) were sequenced using the PacBio RS II platform (Pacific Biosciences, Menlo Park, CA). *De novo* assembly was performed using the Hierarchical Genome Assembly Process version 2 (13). Four circular contigs representing one chromosome (2,927,257 bp; G+C content, 46.59%; coverage, 715.08×) and three plasmids, pEG9A (79,815 bp; G+C content, 43.67%; coverage, 763.18×), pEG9B (55,299 bp; G+C content, 43.10%; coverage, 790.37×), and pEG9C (12,035 bp; G+C content, 40.61%; coverage, 1,408.69×), were obtained.

The numbers of putative coding sequences (CDSs) predicted and annotated by PGAP were 3,025, 102, 72, and 13 for the chromosome, pEG9A, pEG9B, and pEG9C, respectively. Identification using BLAST revealed that the analyzed chromosome was closely related to the chromosome of L. paracasei subsp. paracasei JCM 8130^T^ (query cover, 89%; identity, 99%; GenBank accession number AP012541) and that pEG9C was closely related to pLBC-2 (query cover, 89%; identity, 100%; GenBank accession number AP012543). pEG9A and pEG9B showed partial homology to the L. paracasei N1115 plasmid (query cover, 49%; identity, 96%; GenBank accession number CP007124) and the L. casei LC2W plasmid pLC2W (query cover, 22%; identity, 99%; GenBank accession number CP002617), respectively.

The complete genome sequence of L. paracasei EG9 will help in elucidating the mechanisms of free amino acid production during cheese ripening and will provide insight into the diversity in L. paracasei.

### Accession number(s).

The complete genome sequence of L. paracasei EG9 has been deposited in DDBJ/ENA/GenBank under accession numbers CP029546 (chromosome), CP029547 (pEG9A), CP029548 (pEG9B), and CP029549 (pEG9C).
